# Endosomes as central hubs of interorganellar communication and cellular homeostasis

**DOI:** 10.1093/procel/pwag018

**Published:** 2026-03-18

**Authors:** Yao Feng, Bo Zhou, Zihan Lu, Muyin Guo, Shaohui Zhang, Songlin Wang, Mo Chen

**Affiliations:** Laboratory of Oral Homeostatic Medicine, School of Medicine and SUSTech Homeostatic Medicine Institute (SHMI), Southern University of Science and Technology, Shenzhen 518055, China; Department of Pharmacology, Joint Laboratory of Guangdong-Hong Kong Universities for Vascular Homeostasis and Diseases, School of Medicine and SUSTech Homeostatic Medicine Institute (SHMI), Southern University of Science and Technology, Shenzhen 518055, China; Laboratory of Oral Homeostatic Medicine, School of Medicine and SUSTech Homeostatic Medicine Institute (SHMI), Southern University of Science and Technology, Shenzhen 518055, China; Department of Pharmacology, Joint Laboratory of Guangdong-Hong Kong Universities for Vascular Homeostasis and Diseases, School of Medicine and SUSTech Homeostatic Medicine Institute (SHMI), Southern University of Science and Technology, Shenzhen 518055, China; Laboratory of Oral Homeostatic Medicine, School of Medicine and SUSTech Homeostatic Medicine Institute (SHMI), Southern University of Science and Technology, Shenzhen 518055, China; Department of Pharmacology, Joint Laboratory of Guangdong-Hong Kong Universities for Vascular Homeostasis and Diseases, School of Medicine and SUSTech Homeostatic Medicine Institute (SHMI), Southern University of Science and Technology, Shenzhen 518055, China; Laboratory of Oral Homeostatic Medicine, School of Medicine and SUSTech Homeostatic Medicine Institute (SHMI), Southern University of Science and Technology, Shenzhen 518055, China; Department of Pharmacology, Joint Laboratory of Guangdong-Hong Kong Universities for Vascular Homeostasis and Diseases, School of Medicine and SUSTech Homeostatic Medicine Institute (SHMI), Southern University of Science and Technology, Shenzhen 518055, China; Department of Stomatology, South China Hospital, Medical School, Shenzhen University, Shenzhen 518116, China; Laboratory of Oral Homeostatic Medicine, School of Medicine and SUSTech Homeostatic Medicine Institute (SHMI), Southern University of Science and Technology, Shenzhen 518055, China; Laboratory of Oral Health and Homeostatic Medicine, School of Stomatology and Beijing Laboratory of Oral Health, Capital Medical University, Beijing 100070, China; Department of Biochemistry and Molecular Biology, Capital Medical University School of Basic Medicine, Beijing 100069, China; Laboratory of Oral Homeostatic Medicine, School of Medicine and SUSTech Homeostatic Medicine Institute (SHMI), Southern University of Science and Technology, Shenzhen 518055, China; Department of Pharmacology, Joint Laboratory of Guangdong-Hong Kong Universities for Vascular Homeostasis and Diseases, School of Medicine and SUSTech Homeostatic Medicine Institute (SHMI), Southern University of Science and Technology, Shenzhen 518055, China

**Keywords:** endocytosis, endosome, membrane contact sites, interorganellar communication, cellular homeostasis

## Abstract

Endocytosis mediates the internalization of extracellular cargo via vesicular trafficking, enabling targeted delivery to specific organelles and maintaining cellular homeostasis. Endosomes function as dynamic hubs that orchestrate intracellular communication. While they primarily internalize material from the cell surface, their role extends far beyond passive transport. Through continuous cycles of sorting, fusion, and fission, endosomes engage in extensive crosstalk with other organelles via three fundamental modes: vesicle-mediated cargo transport, membrane contact site-mediated non-vesicular exchange, and signaling and mechanical coupling. These interorganellar interactions enable the transfer of metabolites, lipids, and signals, positioning endosomes as central regulators of cellular homeostasis. While interest in these contacts is growing, a systematic understanding of their roles is still needed. This review explores the protein machinery involved, examines how endosome-organelle contacts coordinate transport and remodeling, and discusses their impact on homeostasis and disease when dysregulated. We underscore the importance of the endosomal communication network in adaptive responses and provide perspectives for targeting endosome-related pathologies.

## Introduction

The plasma membrane’s dynamic nature is vital for cellular homeostasis (Renne and Ernst, 2023). Within this system, endosomes serve as central hubs for membrane trafficking, orchestrating the sorting, recycling, and degradation of both newly synthesized and internalized macromolecules, including membrane proteins, lipids, and carbohydrates (O’Sullivan and Lindsay, 2020). Changes in endosomal lipid composition affect the distribution of associated proteins and the functionality of endosomes (Harayama and Riezman, 2018). Notably, internalized signaling receptors undergo fate determination within endosomes, where their recycling to the membrane or degradation through lysosomal pathways directly modulates signal transduction outcomes, thereby enabling the modulation of dynamic cellular responses (Villaseñor et al., 2016). Thus, endosomes are critical for balancing the reuse and degradation of membrane components, thereby sustaining signal transduction, homeostasis, and cellular function (Chen et al., 2018, 2025b; Hou et al., 2025c; Thapa et al., 2020, 2024). This positions them at the core of the emerging concept of cellular-level homeostatic medicine and highlights their potential as therapeutic targets when dysregulated (Li et al., 2024b, 2025; Wang and Qin, 2022; Zhou et al., 2025).

The endosomal system comprises distinct functional compartments with specialized roles and is categorized into various groups based on their specific functions and characteristics (Endo et al., 2024). During their maturation, endosomes exhibit a sophisticated functional division of labor, centered on the precise distinction between recycling and degradation pathways for cargo fate (Naslavsky and Caplan, 2018, 2020). The early endosomes (EEs) serve as the primary sorting station, which, via its characteristic tubulovesicular morphology, dispatches endocytosed cargo to different destinations: some cargo (such as receptors) can return to the plasma membrane via recycling endosomes (REs), while another portion is sent back to the Golgi apparatus through retrograde transport (Rennick et al., 2021). Meanwhile, the vacuolar domains of EEs generate intraluminal vesicles (ILVs) through budding, which gradually mature into multivesicular bodies (MVBs), representing the morphological hallmark of late endosomes (LEs) (Bissig and Gruenberg, 2013). LEs ultimately fuse with lysosomes, delivering their encapsulated, degradation-destined cargo to the lysosomes for digestion (Cullen and Steinberg, 2018). Collectively, from the sorting decisions made in early endosomes to the packaging and delivery of degradation-targeted cargo by LEs/MVBs, the endosomal system forms an integrated membrane trafficking network essential for cellular homeostasis (Scott et al., 2014).

**Figure 1.**
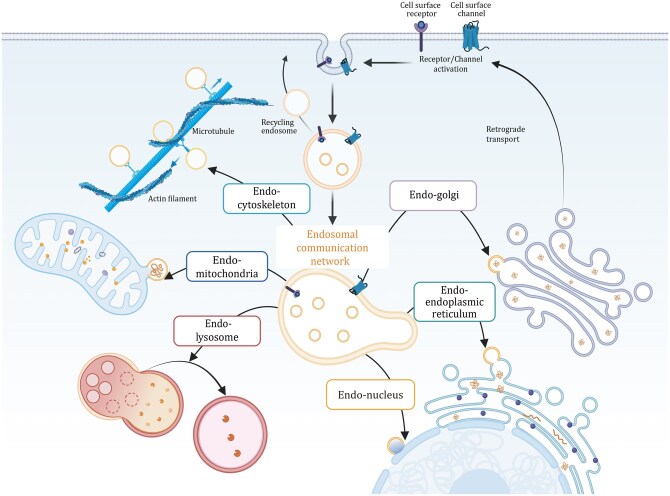
**Overview of endosomal communication network with subcellular components**. Endosomes establish functional connections with various subcellular entities, including the cytoskeleton (microtubules and actin filaments), mitochondria, lysosomes, the nucleus, the endoplasmic reticulum, and the Golgi apparatus. This schematic illustrates the dynamic interactions initiated by receptor/channel activation at the cell surface, which trigger endocytic events. This figure was created using BioRender.

Endosomes are key components of the endomembrane system, facilitating the exchange of membrane components with the plasma membrane and communicating with other organelles. They interface with lysosomes for degradation, collaborate with the Golgi for protein recycling, and interact with the endoplasmic reticulum (ER) for lipid exchange (Mulligan et al., 2023; Tu et al., 2020). Emerging evidence reveals expanded functional connections, including interactions with mitochondria that regulate metabolic coordination, quality control, and organelle dynamics, as well as with nuclear membranes that may modulate gene expression (Naslavsky and Caplan, 2020; Soubannier et al., 2012). These complex interactions depend critically on cytoskeletal networks that provide structural support and directional guidance for vesicle movement (Hou et al., 2025a). The dynamic equilibrium of endosomal compartments and their interorganellar communications has a significant impact on cellular signaling networks. This review examines the endosomal system and how endosomal dynamics modulate specific signaling pathways, highlighting their implications for both cellular physiology and pathophysiology (Fig. 1). To provide a coherent framework for this discussion, we organize our analysis around three fundamental modes of interorganellar crosstalk: (i) vesicle-mediated cargo transport; (ii) membrane contact site (MCS)-mediated non-vesicular communication; and (iii) signaling and mechanical coupling.

## Luminal pH as a key determinant of endosomal homeostasis, identity, and transport

The endosome functions as a central hub in cellular homeostasis, whose activity is profoundly influenced by the proton concentration (pH), which serves as a critical physicochemical switch that dictates the direction of membrane traffic and the nature of interorganellar interactions. The transition from EEs to LEs is characterized by a progressive acidification gradient. EEs maintain a mildly acidic pH of ∼6.5, which is sufficient to trigger the dissociation of many receptor-ligand complexes, enabling receptor recycling. As endosomes mature, increased V-ATPase activity progressively lowers the luminal pH to ∼5.5 in LEs and ∼4.5 in lysosomes. This acidification is not merely a consequence of maturation but an active driver of the process (Hu et al., 2015).

### pH as a temporal signal for Rab GTPase cascades

The precise temporal sequence of Rab GTPase recruitment—the “Rab cascade”—is tightly coupled to this acidification gradient. The drop in pH acts as a permissive signal for the replacement of early Rab5 with late Rab7 (Laifenfeld et al., 2007). The mildly acidic pH of EEs maintains the activity of Rab5 and its effectors, promoting homotypic fusion and cargo sorting. Upon further acidification, pH-sensitive sensors such as the RING finger protein 13 (RNF13) or the V-ATPase itself undergo conformational changes that trigger the recruitment of the Rab7 complex. This ensures that Rab7 acquisition occurs only when the endosome has reached the appropriate maturation stage, preventing premature interaction with dynein motors or lysosomal fusion machinery. As mentioned, some membrane receptors, along with membrane-bound lipids, are transferred to recycling endosomes, returning to the plasma membrane, and this process is regulated by Rab4 and Rab11 (Hsu and Prekeris, 2010; Li and DiFiglia, 2012). The V-ATPase also interacts directly with the regulator complex and amino acid sensors, linking luminal pH to mTORC1 signaling from the lysosomal surface, thereby coordinating degradative capacity with cellular metabolic state.

### pH-dependent recruitment of motor proteins

The acidification state also indirectly influences motor protein recruitment. The recruitment of dynein-dynactin via the Rab7 effector RILP is strictly dependent on the endosome having reached a late-stage pH. Conversely, the recycling machinery (e.g., kinesins bound to retromer) is typically associated with the less acidic, early/recycling endosome compartments (Fares et al., 2025; Renard et al., 2018). Thus, the pH gradient ensures spatial segregation of transport machinery: plus-end directed motors (kinesins) operate on peripheral, less acidic endosomes, while minus-end directed motors (dynein) dominate on perinuclear, acidified late endosomes.

### pH dysregulation causes cellular dysfunction

Genetic defects in V-ATPase subunits cause disruption of endosomal pH, which leads to a breakdown of this temporal order (Indrawinata et al., 2023). Alkalinization of endosomes results in failed cargo dissociation. Rab7 activation occurs too early or too late, misdirecting endosomes to incorrect cellular locations (Guerra and Bucci, 2016). Proper contact site formation often requires a correct luminal pH to present the appropriate lipid or protein landscape.

## Endosome–lysosome interactions: vesicle-mediated degradation transport

**Figure 2.**
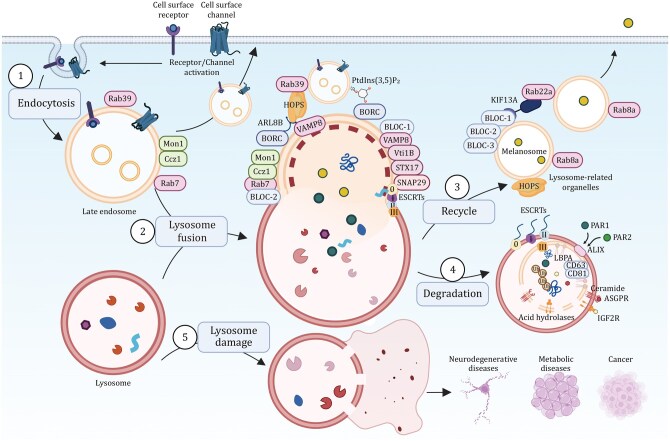
**The endolysosomal pathway and its implications in diseases**. This schematic depicts the sequential stages of endosomal cargo degradation, including lysosomal fusion, intraluminal vesicle (ILV) formation, and post-degradation recycling. (i) Late endosomes fuse with lysosomes, a process regulated by tethering factors including the HOPS complex, RAB7, SNARE proteins, BLOCs, and ESCRT components. (ii) Following fusion, specific molecules are salvaged via Rab-GTPases and BLOCs for reuse. Ubiquitinated cargo is recognized by the ESCRT machinery through a coordinated cascade: ESCRT-0 clusters ubiquitinated cargo, while ESCRT-I and -II drive membrane invagination. ESCRT-III then assembles to constrict and sever the invagination neck, releasing ILVs into the endosomal lumen to form the multivesicular body (MVB). (iii) Multiple alternative pathways also contribute to ILV biogenesis: (a) CD63 and CD81 cluster on endosomal membranes, promoting curvature; (b) Accumulation of LBPA and ceramide induces negative membrane curvature. Subsequently, cargoes including PAR1 and P2Y1 receptors bind directly to ALIX, which interfaces with ESCRT-III to incorporate them into ILVs. Within the acidic lysosomal lumen, hydrolytic enzymes degrade cargo into molecular components. (iv) Lysosome dysfunction compromises degradation efficiency and is implicated in neurodegenerative diseases, metabolic diseases, and cancer. Precise regulation of these pathways ensures selective degradation and cellular homeostasis. Abbreviations: BLOC-1/2/3: biogenesis of lysosome-related organelles complexe-1/2/3; HOPS: homotypic fusion and protein sorting; ESCRTs: endosomal sorting complexes required for transports; ALIX: ALG-2-interacting protein X; Mon1-Ccz1: Morphogenesis and vesicle transport Nucleator 1-Calcium-coupled zinc transporter 1; BORC: BLOC-1-related complex; PtdIns(3,5)P_2_: phosphatidylinositol-3,5-bisphosphate; STX17-Vit1B-VAMP8: syntaxin 17-vesicle transport through interaction with T-SNAREs 1B-vesicle associated membrane protein 8; KIF13A: kinesin family member 13A; IGF2R: insulin-like growth factor 2 receptors; ASGPR: asialoglycoprotein receptor; ARL8B: ARF Like GTPase 8B; Rab7: Ras-related protein Rab-7; Rab8a: Ras-related protein Rab-8A; Rab22a: Ras-related protein Rab-22A; PAR1/2: Protease-Activated Receptor 1/2; LBPA: lysobisphosphatidic acid. This figure was created using BioRender.

Lysosomes are the principal cellular organelles responsible for degrading macromolecules, which are increasingly regarded as metabolic regulatory centers (Singh et al., 2022). Endosomes convey substances, including diverse receptors, lipid membranes, and extracellular fluids, that have been newly internalized via endocytosis. The endolysosomal network is characterized by its interactions, which are crucial for the degradation and recycling of cellular components (Fig. 2) (Cullen and Steinberg, 2018).

### Molecular basis of lysosome fusion with endosomes

LEs fuse with lysosomes through coordinated molecular events to form endolysosomes. The fusion process is regulated by several key components, including the biogenesis of lysosome-related organelle complexes (BLOCs) and homotypic fusion and protein sorting (HOPS) (Li et al., 2007; Raiborg and Stenmark, 2009).

BLOC complexes (BLOC-1, -2, -3) mediate the sorting and transport of specific cargo proteins from EEs to lysosome-related organelles, coordinating degradation and recycling processes by regulating vesicle targeting pathways (More et al., 2024). BLOC-1 is responsible for sorting and transporting specific membrane protein cargoes from EEs to lysosome-related organelles (LROs) (De Pace et al., 2025; Setty et al., 2007). BLOC-2 functions in concert with BLOC-1 and participates in transporting cargoes from late endosomes to organelles such as melanosomes (Di Pietro et al., 2006; Shakya et al., 2018). BLOC-3, by activating Rab GTPases (Rab32/38), ensures that transport vesicles can precisely target and fuse with the membrane of terminal organelles like melanosomes (Dennis et al., 2016). MON1-CCZ1 (Morphogenesis and vesicle transport Nucleator 1-Calcium-coupled zinc transporter 1) complex acts as the exclusive guanine nucleotide exchange factor (GEF) for Rab7. It activates Rab7-GTP on EEs and recruits the HOPS complex to promote the fusion of LEs with lysosomes, enabling content degradation (Klink et al., 2022). The BLOC-1-related complex (BORC) shares subunits with the BLOC-1 complex, such as Snapin and dysbindin, and is a complex that regulates lysosome positioning and movement, acting upstream of ARL8B (ARF-like GTPase 8B) (Pu et al., 2015). BORC not only directly governs the endosome pool size through PIKfyve-dependent phosphatidylinositol-3,5-bisphosphate (PtdIns(3,5)P_2_) production, but also further mediates the reverse transport of LEs to lysosomes through shared subunit interaction (Di Giovanni and Sheng, 2015; Yordanov et al., 2019). Furthermore, the soluble N-ethylmaleimide-sensitive factor attachment protein receptor (SNARE) complex, composed of Syntaxin 7 (STX7), Syntaxin 8 (STX8), Vti1B, and Vesicle-associated membrane protein 7 (VAMP7), mediates the heterotypic fusion between LEs and lysosomes (Luzio et al., 2009).

HOPS is a heterohexameric complex whose subunits have different functions regarding protein localization and effector binding, such as vacuolar protein sorting 11 (Vps11), Vps41, Vps16, etc., which precisely mediates the localization of multivalent membrane-binding factors and a SNARE chaperone to lysosomes (Peplowska et al., 2007; van der Kant et al., 2015). Both Vps41 and Vps39 contain a Rab-binding subunit and anchor HOPS to the surface of lysosomes (Bröcker et al., 2012). In higher eukaryotes, HOPS is also suspected to bind ARL8B via Vps41 (Marwaha et al., 2017). The HOPS complex recruits TBC1D15 to catalyze a Rab7-to-ARL8B GTPase switch on late endosomes (LEs), inactivating Rab7 while activating ARL8B on lysosomal membranes (Jongsma et al., 2020). Vps11 and Vps18 connect the HOPS head and tail, maintaining the structural stability of the complex and promoting the formation of multivalent membrane tethering (Nickerson et al., 2009; Plemel et al., 2011). The Vps41-Vps16-Vps18-Vps33A subcomplex function regulates autophagosome-lysosome fusion (Zhang et al., 2024). Vps33, an S/M-family protein that chaperons SNARE assembly, conformed to activate the binding of the lysosomal membrane SNARE protein synaptosome-associated protein 29 kDa (SNAP29) and the LEs membrane STX17-VAMP8, conformed to activate the SNARE protein, drive the assembly of the trans-SNARE complex, and finally complete the fusion of endosomes and lysosomes through membrane lipid recombination to form fusion pores (Baker et al., 2015). BORC knockout reduces the recruitment of HOPS to lysosomes and the assembly of the trans-SNARE complex, comprising STX17, VAMP8, and SNAP29 (Itakura et al., 2012; Jia et al., 2017; Mullock et al., 2000; Pryor et al., 2004). HOPS ensures fusion efficiency and specificity through the dual effects of tethering and SNARE activation. The Vps33A subunit is correctly recruited to the HOPS complex through specific interactions with the Vps16 subunit, catalyzing the proper assembly of SNARE proteins. If the binding between Vps16 and Vps33A is artificially disrupted, the HOPS complex cannot rescue endosome-lysosome fusion (Graham et al., 2013; Wartosch et al., 2015).

### Physiological function of degradation or recirculation pathways

During LE maturation, ubiquitinated membrane proteins were sorted into intraluminal vesicles (ILVs) by the endosomal sorting complexes required for transport (ESCRT) machinery for lysosomal degradation (Luzio et al., 2009) Once fusion occurs, the contents of the LEs, including luminal vesicles and cargo proteins, are transported to lysosomes. ESCRT-0 aggregates ubiquitinated cargo, while the ESCRT-I subunit further enriches it through low-affinity binding to ubiquitin (Banjade et al., 2022; Migliano et al., 2024). ESCRT-II senses the cargo density and recruits ESCRT-III. ESCRT-III oligomerizes to control the cargo, limit the lateral diffusion on the endosomal membrane, and promote the germination of ILVs (Teo et al., 2004). However, not all ILV formation depends on the ESCRT complex; other mechanisms are equally important. Transmembrane protein families such as CD63 and CD81 can form microdomains on the endosomal membrane, promoting membrane curvature and ILVs formation (Theos et al., 2006; van Niel et al., 2011). This mechanism is particularly critical in specialized organelles, such as melanosomes. Additionally, changes in the lipid composition of the endosomal membrane, such as lysobisphosphatidic acid (LBPA) or ceramide, can induce negative membrane curvature, driving ILV budding independently of the ESCRT machinery (Bissig and Gruenberg, 2013). Some cargo (such as PAR1 and P2Y1 receptors) bypass ubiquitination and enter the lysosome for degradation by directly binding to ALIX (ALG-2-interacting protein X), which interacts with ESCRT-III (Dores et al., 2012, 2016; Teo et al., 2004). On the other hand, endosome–lysosomal interactions ensure that receptors are effectively transported to lysosomes for degradation (Bright et al., 2016). For instance, insulin-like growth factor 2 receptors (IGF2R), asialoglycoprotein receptor (ASGPR), and sortilin were demonstrated to incorporate receptor tags that fuse into soluble or transmembrane target protein complexes, enabling lysosomal trafficking and subsequent degradation. This occurs through IGF2 binding, which induces receptor dimerization and conformational changes, directing the complexes to lysosomes for proteolysis (Huang et al., 2025). This process is crucial for preventing excessive signaling pathway activation and maintaining cellular homeostasis.

Furthermore, Rab-GTPases play a crucial role in regulating the recycling pathway. Rab8a controls secretory autophagy (such as the secretion of α-synuclein), which promotes an alternative pathway (secretory autophagy) that diverts specific cargo away from the default degradative pathway (Lin et al., 2024). BLOC can recycle cargo to the cell surface and transport cell-type-specific cargo to lysosome-related organelles, such as melanosomes in melanocytes (Zhang et al., 2014). Rab22a forms complexes with the BLOC-1, BLOC-2, and driver protein-3 family motor kinesin family member 13A (KIF13A) on the endosome to promote cargo recycling. Knockout of the Rab22a gene leads to a decrease in recirculation kinetics and simultaneously causes cargo accumulation in endosomes or lysosomes (Shakya et al., 2018). Additionally, the deletion of HOPS complexes (Vps11, Vps39, and Vps41) disrupts the early to late endosome transformation, impairs the functions of endosome recycling, and induces endosome accumulation (van der Beek et al., 2024).

### Pathological implications

The interaction between the endosome and the endolysosomal enzyme system is crucial for maintaining cellular homeostasis. Mutations in key proteins of the endolysosomal system can cause lysosomal functional defects and lead to various diseases. In lysosomal storage disorders, genetic studies have linked gene mutations encoding lysosomal enzymes or regulatory proteins (such as Rab GTPases or HOPS subunits), membrane proteins, or transport mechanisms that usually lead to endosome-lysosome fusion defects (Pagano, 2003; Zhang et al., 2017). Furthermore, mutations in HPS genes cause Hermansky-Pudlak syndrome (HPS). The majority of these genes, including HPS1, HPS3, HPS4, HPS5, HPS6, HPS7, and HPS8, encode subunits of the biogenesis of LROs Complexes (BLOCs) (Di Pietro et al., 2004). For example, BLOC-3 is a protein complex containing the HPS gene products HPS1 and HPS4. Functional assays using zebrafish models confirm the significance of BLOC-3 assembly and its interaction with Rab9a during melanosome biogenesis (Martina et al., 2003; Yong et al., 2025). Mutations in the Vps genes (Vps16 and Vps39) disrupt lysosomal fusion mediated by the HOPS complex in zebrafish models, leading to the aggregation of β-amyloid protein and α-synuclein, which are respectively involved in the pathological processes of Alzheimer’s disease (AD) and Parkinson’s disease (Banerjee et al., 2024; Li et al., 2024a).

The dysregulation of the degradation/recycling pathway can lead to the accumulation of undegraded substances, thereby triggering cancer progression and metabolic diseases. Evidence from cancer cell models suggests that defects in autophagy-lysosomal flow may lead to the release of carcinogenic proteins (such as mutant p53), potentially accelerating metastasis and immune evasion (Chang and Dong, 2020). Preclinical study suggests the deficiency of phosphatidylinositol 4-kinase type 2 alpha/beta (PI4K2A/B) leads to the misclassification of BLOC-1 dependent cargo (such as tyrosinase), resulting in impaired melanin synthesis and hypopigmentation of the skin (Zhu et al., 2022). Defects in the BORC-ARL8B-HOPS pathway may lead to perinuclear retention of LEs and lipofuscin accumulation, both of which are closely related to cancer cell invasion (Wu et al., 2020). Additionally, sortilin—an endosomal protein—mediates the trafficking of ACSL1 from mitochondria to endosomes for proteolytic clearance, consequently inhibiting fatty acid oxidation and thermogenic function in adipocytes of male mice (Yang et al., 2024). In atherosclerosis, preclinical studies suggest that cholesterol crystal-induced macrophage lysosomal rupture exemplifies this chronic inflammatory trigger (Wang et al., 2025). Once endosome-lysosome contacts are established, aberrant transport of material disrupts lysosomal homeostasis. It impairs degradative capacity, causing a backlog of damaged mitochondria and protein aggregates (tau, α-synuclein)—a key feature of Parkinson’s disease and AD (Takahashi et al., 2024; Wong and Krainc, 2017). This autophagic stress destabilizes cells, and the released partially digested material seeds further aggregation. Concurrently, cathepsin release, mitochondrial ROS, and potassium efflux activate the NOD-like receptor family pyrin domain-containing 3 (NLRP3) inflammasome (Cai et al., 2022). Caspase-1 maturation of IL-1β and IL-18 drives inflammation hallmarking gout and atherosclerosis (Tanaka et al., 2022).

## Endosome–Golgi apparatus interactions: vesicle-mediated retrograde transport

**Figure 3.**
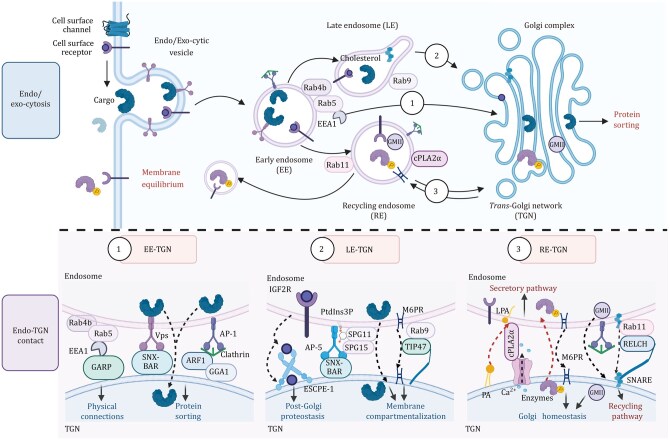
**The endosome-Golgi interaction in cellular transport**. Following cargo internalization via endocytosis, multiple pathways sort proteins from endosomes to the *trans*-Golgi network (TGN). (i) Early endosome (EE) sorting: Rab GTPases (Rab4b/Rab5) in conjunction with the GARP complex mediate EE-TGN membrane contact sites. SNX-BAR adaptor proteins coordinate with clathrin adaptors (AP-1, GGAs, Clathrin, and ARF1) to assemble retrograde transport carriers containing cargo proteins. (ii) Late endosome (LE) transport: the ESCPE-1 complex drives retrograde transport of specific cargoes (e.g., IGF2R), revealing functional hierarchy within endosomal retrieval systems. Rab9 GTPase precisely regulates LE-to-TGN trafficking through GTP-dependent recruitment of effector molecules (TIP47). Also, AP-5 acts in concert with SPG11 and SPG15 to facilitate the recycling of cargo. (iii) Recycle endosome regulation: cPLA2α-mediated rapid secretion promotes tubular transport carrier formation for secondary secretion. The M6PR targets enzymes to destination compartments via specific recognition. Retrograde COPI vesicles retrieve escaped resident enzymes (e.g., GMII), maintaining secretory Golgi compartment homeostasis. Rab11 GTPase coordinates cholesterol transfer from LEs to Golgi membranes through RELCH. The coordinated operation of endosome-TGN and intra-Golgi transport networks ensures precise protein localization, functional execution, and organelle structural integrity. Abbreviations: EEA1: early endosome antigen 1; AP-1: adaptor protein complex 1; AP-5: adaptor protein complex 5; ARF1: ADP-ribosylation factor 1; GGAs: Golgi-localized γ-ear-containing ARF-binding proteins; SPG11: SPG11 vesicle trafficking associated, spatacsin; SPG-15: SPG15 vesicle trafficking associated, spatacsin; SNX-BAR: sorting nexin-bin-amphiphysin-rvs; Vps: vacuolar protein sorting; ESCPE-1: endosomal SNX-BAR sorting complex for promoting exit; IGF2R: insulin-like growth factor 2 receptor; TIP47: tail-interacting protein of 47 kDa; SNARE: soluble N-ethylmaleimide-sensitive factor attachment protein receptors; M6PR: the mannose-6-phosphate receptor; LPA: lysophosphatidic acid; PA: phosphatidic acid; Rab4b: Ras-related protein Rab-4b; Rab5: Ras-related protein Rab-5; Rab9: Ras-related protein Rab-9; Rab11: Ras-related protein Rab-11; cPLA2α: cytoplasmic phospholipase A2α; GMII: Golgi α-mannosidase II; GARP: the Golgi associated retrograde protein complex. This figure was created using BioRender.

The Golgi apparatus is a central eukaryotic organelle responsible for processing, modifying, sorting, and dispatching cellular cargo. It’s organized into a series of flattened, membrane-bound cisternae stacked upon each other, functionally divided into distinct regions: the cis-Golgi network (CGN), the medial cisternae, and the *trans*-Golgi network (TGN) (Pirozzi et al., 2025). The TGN serves as a pivotal sorting hub in cellular trafficking pathways, integrating both endocytic and secretory routes. At this juncture, endocytic material intersects with biosynthetic cargo, retrieving errant Golgi enzymes through retrograde transport while enabling receptor recycling to the plasma membrane. These coordinated processes sustain membrane equilibrium and ensure fidelity in secretory system operations (Fig. 3) (Bonifacino and Glick, 2004). These functions collectively ensure that the endosomal hub maintains productive communication with the Golgi, enabling coordinated regulation of secretion, degradation, and signaling.

### Molecular basis of protein sorting and tethers

Retrograde trafficking is orchestrated by a suite of molecular machines that operate in a coordinated sequence: cargo selection, membrane deformation, carrier formation, and target recognition. Building upon this principle, the biogenesis of selective transport carriers necessitates the precise cytosolic deployment of molecular machinery to specialized endomembrane domains, thereby establishing substrate-specific retrograde trafficking circuits (Johannes and Popoff, 2008; Traub and Bonifacino, 2013). Cargo selection is primarily mediated by adaptor protein complexes that recognize specific sorting motifs on cargo cytoplasmic tails. AP-1 and GGAs (Golgi-localized γ-ear-containing ARF-binding proteins) are recruited to TGN and endosomal membranes by the small GTPase ARF1-GTP and phosphoinositides [e.g., phosphatidylinositol 4-phosphate (PtdIns4P)] (Robinson et al., 2024). These adaptors concentrate cargo and recruit clathrin, which provides a mechanical scaffold for vesicle budding (Daboussi et al., 2012). Unlike AP-1 and GGAs, which operate primarily at the TGN, the retromer complex (Vps26-Vps29-Vps35) functions on endosomes to recognize a distinct set of cargoes, often through indirect interactions mediated by sorting nexins (Seaman, 2021). The receptor recovery vector comprises a tubular structure formed by the retromer complex (Vps26/29/35) and sorting nexin 1/6 (SNX1/6), which transports the mannose-6-phosphate receptor (M6PR) in reverse to the TGN (Seaman, 2004). This mechanism also involves Sortilin-mediated neuroendocrine protein sorting (Kim and Gadila, 2016; Park et al., 2021).

Membrane remodeling is executed by sorting nexin (SNX) family proteins (Yong et al., 2018), many of which contain BAR (Bin-Amphiphysin-Rvs) domains that sense and induce membrane curvature (Peter et al., 2004). SNX1/SNX2 heterodimers partner with SNX5/SNX6/SNX32 to form the ESCPE-1 (endosomal SNX-BAR sorting complex for promoting exit 1) complex (Lopez-Robles et al., 2023), which generates tubular carriers enriched for cargo such as the insulin-like growth factor 2 receptor (IGF2R) (Evans et al., 2020). These SNX-BAR proteins operate in parallel with retromer, with ESCPE-1 driving membrane tubulation while retromer contributes to cargo sorting—a functional stratification that exemplifies the modular design of endosomal retrieval systems (Arighi et al., 2004; Evans et al., 2020; Kvainickas et al., 2017).

### Dynamic regulation of trafficking routes

Tethering and fusion at the TGN are mediated by multisubunit tethering complexes, including the GARP (Golgi-associated retrograde protein) complex, which is recruited by Rab4b and facilitates SNARE-mediated fusion. The first pathway discovered to mediate retrograde transport from an endocytic compartment to the TGN in mammalian cells was not through AP-1 or the retromer complex, but rather through the Rab9/tail-interacting protein of 47 kDa (TIP47) pathway. This process is coordinately regulated by Rab9, a GTPase that governs LE-to-TGN transport by recruiting effector molecules (Pfeffer, 2009). Emerging evidence positions the AP-5 complex as a critical mediator of retrograde cargo retrieval, operating through non-canonical endosome-Golgi trafficking routes to regulate the sorting of IGF2R and sortilin-family receptors (Han et al., 2021; Takeda et al., 2019). Moreover, both the cation-independent mannose 6-phosphate receptor (CIMPR) and sortilin interacted with the AP-5-associated protein spastic paraplegia 15 (SPG15) in pull-down assays, and we propose that sortilin may act as a link between Golgi proteins and the AP-5/SPG11/SPG15 complex (Hirst et al., 2018; Renvoisé et al., 2014). Also, Rab GTPase activation initiates with GEFs triggering GTP loading, enabling Rab proteins to recruit effector complexes, such as early endosome antigen 1 (EEA1) and tethering factors, during EEs maturation (Lamber et al., 2019). The class C core endosomal vacuole tethering (CORVET) complex collaborates with Rab5 to complete tethering (Balderhaar et al., 2013). Rab4b, as an effector of the Golgi-associated retrograde protein complex, maintains the correct localization of the mannose-6-phosphate receptor (M6PR) (Gilleron et al., 2024). Endosome-to-TGN transport not only occurs from tubular EEs but also from LEs. Rab9 specifically regulates M6PR recycling from LEs to the TGN (Kucera et al., 2016). Furthermore, the RELCH protein (containing LisH, coiled-coil, and HEAT repeats) establishes intercompartmental connectivity by binding Rab11 on REs and oxysterol-binding protein (OSBP) on the TGN, thereby coordinating cholesterol transfer from LEs to Golgi membranes (Sobajima et al., 2018). These multilayered regulatory systems highlight the spatiotemporal precision of endosomal trafficking networks.

### Physiological function of retrograde transport

The TGN orchestrates cargo dispatch through two distinct transport modalities mediated by specialized sorting receptors (Park et al., 2021): (i) Constitutive secretory ­retromer complex and retriever complex recognize the dileucine motif, and govern baseline secretion of ­housekeeping proteins (Bowers and Stevens, 2005; Duncan, 2022; Martinez-Menárguez et al., 2001); (ii) utilizes calmodulin-dependent membrane budding machinery and packages dense-core granules through calcium-sensitive lipid remodeling (Vitale et al., 2002).

The cisternal maturation mechanism holds that secretory proteins undergo anterograde transport to the TGN within 20–40 min, while COPI vesicles maintain compartment homeostasis by recovering resident proteins (such as Golgi α-mannosidase II (GMII)) through alternative sorting signals and receptors specific for Golgi-resident enzymes (Rose, 2012). Also, the COPI pathway, through KDEL receptors, plays a pivotal role in ER-resident protein recovery. The newly discovered cytosolic phospholipase A2α (cPLA2α)-mediated rapid secretion pathway hydrolyzes membrane phosphatidic acid (PA) to generate lysophosphatidic acid (LPA), altering the curvature of the lipid bilayer to drive COPI vesicles to form tubular transport channels, which can achieve second-level secretion when calcium signals are activated (Raote and Malhotra, 2019; Yang et al., 2011). Furthermore, transmembrane receptors such as sortilin and M6PR play essential roles in directing soluble lysosomal enzymes to their designated compartments through distinct recognition mechanisms (Braulke and Bonifacino, 2009). This tubular network maintains the integrity of the Golgi apparatus membrane, and cPLA2α knockout can cause structural fragmentation. Concurrently, resident protein localization is maintained through COPI vesicle-mediated retrograde recycling, establishing a dynamic equilibrium between anterograde flux and retrograde retrieval. In addition to retrieving its own resident proteins, the Golgi apparatus depends on endosome-mediated retrograde transport for the recycling of diverse cargoes essential for its functional integrity. These include transport receptors, adaptor proteins involved in vesicle coat assembly, specific SNARE proteins required for membrane fusion, and cell-surface signaling receptors such as G protein-coupled receptors (GPCRs) and receptor tyrosine kinases (RTKs) (McNally and Cullen, 2018; Yong et al., 2022). By retrieving these molecules from endosomal compartments, the retrograde pathway ensures proper protein localization, sustains Golgi function, and maintains cellular homeostasis.

### Pathological implications

Endosomes and the Golgi apparatus interact closely through membrane trafficking, signaling, and membrane contact sites to jointly maintain homeostasis of the endomembrane system. Dysfunction in these interactions is closely associated with the pathophysiology of various diseases: (i) Hermansky-Pudlak syndrome is caused by mutations in multiple genes encoding components of vesicle-mediated retrograde transport, including SPG11, SPG15, and subunits of the AP-5 complex. These proteins function together at LEs to mediate retrograde transport of cargoes, such as IGF2R, to the Golgi. The retromer complex (Vps26/Vps29/Vps35), a key component of vesicle-mediated transport from endosomes to the Golgi, is downregulated in vulnerable neurons in AD brains. Retromer deficiency disrupts the retrieval of the sorting receptor sortilin-related receptor 1 (SORL1) from endosomes to the Golgi, leading to mistrafficking of amyloid precursor protein (APP) into endosomal compartments where it undergoes β-secretase cleavage, generating amyloid-β (Aβ) (Di Pietro et al., 2006); (ii) dysregulated signaling (mTOR-TFEB axis abnormalities) exacerbates lysosomal storage disorders and metabolic reprogramming in diseases from preclinical studies (Gros and Muller, 2023; Napolitano and Ballabio, 2016); (iii) analysis of patient-derived fibroblasts reveals that disruption of membrane contact sites (due to SPG11 mutations) impairs lipid metabolism and autophagy, leading to hereditary spastic paraplegia and Parkinson’s disease (Long et al., 2025; Renvoisé et al., 2014). Neurons are particularly vulnerable to such defects, making the underlying mechanisms critical targets for therapeutic intervention.

## Endosome–ER interactions: MCS-mediated material transport

**Figure 4.**
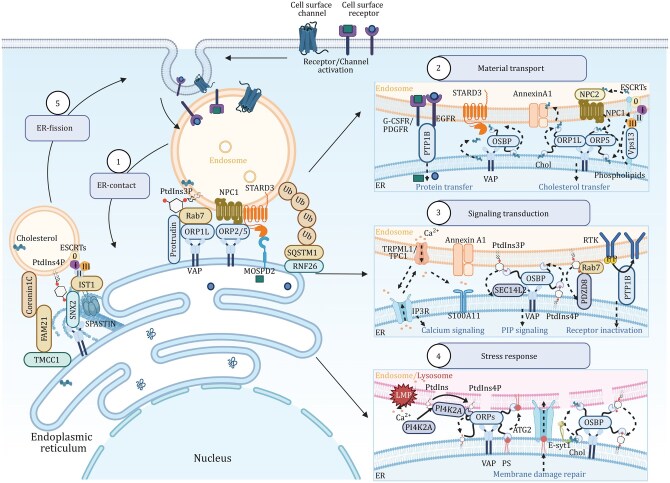
**The endosome-ER communication network and functional interplay**. The molecular and functional interplay between the endoplasmic reticulum (ER) and endosomes is organized into five core processes essential for inter-organelle communication and cellular homeostasis. (i) Endosome-ER contact: ER-endosome contact is initiated by receptor/channel activation (e.g., cell surface receptors), triggering endocytosis. The Protrudin-Rab7 complex, the VAP-ORPs complex, the ubiquitination complex, and MOSPD2 bind motifs drive membrane contraction to stabilize ER-endosome contact sites. (ii) Material transport: EGFR, G-CSFR, and PDGFR are transported between the endosome-ER contact through PTP1B. ER-to-endosome cholesterol transport is mediated by STARD3 (via VAP tethering), with OSBP. Additionally, cholesterol recycling to endosomes occurs via VAP-ORP1L/ORP5 complexes, which are stabilized by Annexin A1, while NPC1/NPC2 extracts luminal cholesterol from endosomes for ER transport. The bridge-like protein Vps13 mediates lipid transfer in the ESCRT pathway. (iii) Signaling transduction: Endosomal Ca^2+^ release occurs through TRPML1/TPC1 channels, while ER Ca^2+^ efflux is mediated by IP3 receptors. The S100A11-Annexin A1 heterotetramer stabilizes subcellular Ca^2+^ microdomains, extending signal duration to synchronize organelle responses. At ER-endosome contact sites, SEC14L2 enables OSBP/ORPs (tethered via VAP-A/B) to dock and transfer PtdIns4P/PtdIns3P. Additionally, PDZD8-Rab7 interactions drive PtdIns4P transfer from the ER to endosomes. Furthermore, PTP1B (localized at ER membranes) inactivates RTK through dephosphorylation. (iv) Stress response: LMP triggers Ca^2+^-dependent recruitment of PI4K2A, generating elevated PtdIns4P on damaged lysosomes. PtdIns4P recruits ORP9/10/11 to establish ER-lysosome contacts, mediating ER-to-lysosome PS transport. Lysosomal PS accumulation activates ATG2, delivering lipids for direct membrane repair. Parallel to ORPs, OSBP is a redundant tether that transports cholesterol (not PS) to damaged sites. E-Syt1 is another critical tethering protein recruited by PtdIns4P. (vi) ER fission is executed by the ESCRT-Spastin complex, with Coronin1C and IST1 mediating membrane scission. TMCC1 stabilizes pre-fission ER structures. SNX2 and FAM21 recruit ESCRT and activate Coronin1C/IST1 to orchestrate fission. Abbreviations: EGFR: epidermal growth factor receptor; G-CSFR: granulocyte colony stimulating factor receptor; PDGFR: platelet-derived growth factor receptors; PtdIns3P: phosphatidylinositol-3-phosphate; PtdIns4P: phosphatidylinositol-4-phosphate; PTP1B: ER-resident protein protein-tyrosine phosphatase 1B; SEC14L2: SEC14-like lipid binding protein 2; VAP: vesicle-associated membrane protein-associated protein; OSBP: oxysterol-binding protein; PS: phosphatidylserine; Annexin A1: calcium-dependent phospholipid-binding protein; NPC1: Niemann-Pick C1; NPC2: Niemann-Pick C2 protein; ORP1L: OSBP-related protein 1L; ORP5: OSBP-related protein 5; STARD3: STAR-related lipid transfer domain containing 3; PDZD8: PDZ domain containing 8; IP3R: Inositol 1,4,5-trisphosphate receptor; TRPML1: transient receptor potential mucolipin subfamily member 1; TPC1: Two-pore channel protein 1; S100A11: S100 calcium binding protein A11; RNF26: ring finger protein 26; SQSTM1: Sequestosome 1; ESCRTs: endosomal sorting complex required for transports; IST1: increased sodium tolerance1; SNX2: sorting nexin 2; TMCC1: transmembrane and coiled-coil domain protein 1; FAM21: family with sequence similarity 21 member; Rab7: Ras-related protein 7; RTK: tyrosine kinase; LMP: lysosomal membrane permeabilization; MOSPD2: motile sperm domain-containing protein 2; ATG2: autophagy related 2 homolog A; E-Syt1: extended synaptotagmin-1; Vps13: vacuolar protein sorting 13; ESCRTs: endosomal sorting complexes required for transports; PI4K2A: phosphatidylinositol 4-kinase type 2 alpha. This figure was created using BioRender.

The ER, the cell’s largest organelle, forms a communication network with almost all other organelles. Notably, interactions with endosomes increase as endosomes mature and transport cargo, with LEs playing a key role in structuring this ER communication network (Friedman et al., 2013; Wenzel et al., 2022). These ER-endosome contact sites are essential for various cellular processes, including endosome dynamics, lipid transport, and calcium signaling (Fig. 4).

### Molecular basis of ER-endosome contact sites

At least seven functionally specialized membrane contact sites (MCSs) between the ER and endosomes have been identified. These dynamic structures, maintained by molecular synergies, preserve compartmental characteristics and regulate membrane trafficking. The OSBP and its related protein (ORP) families play a central role in this regulation. OSBP, anchored at LE-ER contact sites via an FFAT motif, mediates lipid exchange between compartments (Olkkonen and Ikonen, 2022; Rocha et al., 2009). The long isoform of OSBP-related protein lL (ORP1L), in tandem with Rab7, interacts with ER membrane VAMP-associated proteins via an FFAT motif, thereby regulating endosome movement (Johnson et al., 2016; Rocha et al., 2009; van der Kant et al., 2013). ORP2 and ORP5, as ER-anchored lipid transfer proteins, bind to Niemann-Pick C1 protein (NPC1) at specific membrane contact sites, thereby enhancing ER–LE interactions (Du et al., 2020; Hynynen et al., 2005; Wang et al., 2019). When endosome-lysosome contacts or NPC protein function is abnormal, cholesterol efflux is blocked, leading to its massive accumulation in the lysosome. Experimental models show that this not only causes Niemann-Pick type C disease but is also closely related to the occurrence and development of hypercholesterolemia and atherosclerosis (Wang et al., 2025). Similar to ORP1L, protrudin, an ER membrane protein with an FFAT motif, dynamically induces contact site assembly by sensing Rab7-GTP states and PtdIns3P distribution (Pedersen et al., 2020; Raiborg et al., 2015). Ring Finger Protein 26 (RNF26), an ER-anchored E3 ubiquitin ligase, regulates protein recruitment to endosomal compartments through Sequestosome 1 (SQSTM1) ubiquitination (Cremer et al., 2021; Jongsma et al., 2016; Liang et al., 2021). Motile sperm domain-containing protein 2 (MOSPD2) binds FFAT motifs and consequently allows membrane tethering *in vitro*. MOSPD2 is an ER-anchored protein, and it interacts with several FFAT-containing tether proteins from endosomes (Di Mattia et al., 2018).

MCSs play a critical role in the spatiotemporal coordination of endosome fission, influencing endosome maturation and cargo sorting through a multi-level regulatory network (Rowland et al., 2014). Coronin 1C and transmembrane and coiled-coil domain family 1 (TMCC1) mediate the recruitment of ER tubules to endosome fission sites marked by family with sequence similarity 21 (FAM21); the absence of either protein leads to fission defects (Hoyer et al., 2018; Striepen and Voeltz, 2022). The inclusion of increased sodium tolerance 1 (IST1) into the ESCRT complex allows the recruitment of spastin and controls membrane reshaping at ER-endosome contact sites through tubulin cutting, providing mechanical force for fission (Allison et al., 2013, 2017). Synergizing with SNX2, VAP regulates PtdIns4P metabolism to maintain fission site membrane properties (Dong et al., 2016).

### Physiological function of material transport

MCSs help sort cargo proteins into transport vesicles during endosomal trafficking pathways (Prinz et al., 2020). Malfunctions in MCS can lead to disrupted transport pathways. The dynamic interaction between Rab7-positive endosomes and the ER forms the structural framework of the transport network, directly impacting compartment morphology and functional homeostasis (Di Mattia et al., 2020; Rowland et al., 2014; Wang et al., 2024). Rab9a utilizes an FSV region to recruit Reticulon (Rtn3L) via its six LC3-interacting region motifs. This complex regulates endosome maturation and cargo sorting (Wu and Voeltz, 2021). Leucine-rich repeat kinase 1 (LRRK1) phosphorylates Rab7 at S72, forming a “phosphorylated Rab7-RILP-dynamin” cascade that mediates the directional transport of the epidermal growth factor receptor (EGFR) from endosomes to the perinuclear region, revealing a phosphorylation-dependent cargo-sorting mechanism (Hanafusa et al., 2019). The ER-resident protein-tyrosine phosphatase 1B (PTP1B) plays a role in MCSs by dephosphorylating endocytosed EGFR, coordinating signal attenuation and lysosomal degradation. It also regulates the transport and sorting of granulocyte colony-stimulating factor receptor (G-CSFR) and platelet-derived growth factor receptor (PDGFR) (Eden, 2016; Eden et al., 2010).

The establishment of a specific lipid microenvironment at ER-endosome contact sites, critical for membrane fission, is mediated by coordinated lipid transfer. SEC14L2 (SEC14 Like Lipid Binding 2) localizes to Golgi-derived vesicles within these contacts and facilitates PtdIns4P transfer, thereby regulating the recruitment of PtdIns4P-effectors (e.g., OSBP, ORPs) to enable fission (Gong et al., 2021). Simultaneously, ORP10-tethered via ORP9 and vesicle-associated membrane protein (VAP)-operates at these sites to supply phosphatidylserine (PS) to endosomes in return for PtdIns4P (Kawasaki et al., 2022). Additionally, the bridge-like protein Vps13 mediates non-vesicular lipid transfer at these contact sites, a process required for the ESCRT pathway (Suzuki et al., 2024).

### Physiological function of lipid metabolism and calcium signaling

ER contact sites are crucial for lipid transfer within cells, particularly for cholesterol and phosphoinositides, such as PtdIns4P. OSBP and ORPs play a key role in exchanging cholesterol and PtdIns4P between the ER and endosomes, thereby achieving counter-exchange (Tan and Finkel, 2022). ORP1L-deficient cells exhibit cholesterol transfer defects, such as reduced ER cholesterol esterification, increased sterol regulatory gene expression, cholesterol and fatty acid biosynthesis, and cholesterol accumulation on endosomes (Vihervaara et al., 2011; Zhao and Ridgway, 2017). Deprivation of LDL-cholesterol induces ORP1L recruitment to ER-MVB contact sites marked by annexin A1, promoting ER-derived cholesterol transfer to MVBs and intraluminal vesicle formation (Eden et al., 2016). ORP5 and NPC1/2 deficiencies also accumulate cholesterol on LE membranes (Luo et al., 2019). ORP8, a homolog of ORP5, is anchored in the ER via its C-terminal transmembrane segment and acts as a multifunctional sterol sensor at the ER and nuclear membranes. However, its function in endosomes remains unconfirmed (Zhao and Ridgway, 2017). In addition to ORPs/VAP contacts, StAR-related lipid transfer domain-containing 3 (STARD3) has also been shown to mediate cholesterol transfer from the ER to LEs at ER-LE contact sites (Wilhelm et al., 2017). Overexpressed STARD3 redirects newly synthesized cholesterol to LEs, reducing plasma membrane cholesterol levels. PDZD8 (PDZ Domain Containing 8), an ER-intrinsic membrane protein with an SMP domain family lipid transfer module, interacts with GTP-Rab7 to mediate lipid transport (Eden, 2016).

The ER membrane system regulates calcium signaling and the activity of growth factor receptors. MSCs facilitate the exchange of calcium ions and signaling molecules, influencing endosome function and cellular responses. Like the ER, endosomes serve as Ca^2+^ storage organelles, releasing Ca^2+^ through TRPML1 (transient receptor potential mucolipin 1) and TPC1 (two-pore cation channel 1) (Kilpatrick et al., 2013). ER-endosome contacts are regulated by local Ca^2+^ release from endosomes, with TPC1 playing a key role at these sites. TPC1 deficiency reduces contact frequency and impedes EGFR-PTP1B interaction. Evidence suggests Ca^2+^ released from the ER via IP3R (inositol 1,4,5-trisphosphate receptor) may be sequestered into endosomes and lysosomes through unknown channels (López-Sanjurjo et al., 2013). Annexin A1 forms a heterocomplex with the calcium-sensing protein S100A11 (S100 Calcium Binding Protein A11), bridging the ER and EGFR-positive endosomes through calcium-dependent conformational changes to establish specialized MCS structures for transport (Eden et al., 2016). Despite identifying various ER contact sites with unique molecular structures and functions, further research is needed to understand their formation, functional mechanisms, and how they coordinate cellular functions.

### Pathological implications of stress response

Following lysosomal membrane permeabilization (LMP), the released Ca^2+^ triggers the recruitment of PI4K2A to generate a local pool of phosphatidylinositol 4-phosphate (PtdIns4P) on the damaged membrane (Tan and Finkel, 2022). This PtdIns4P acts as a recruitment signal for ORP9, ORP10, and ORP11, which establish tight membrane contact sites between the ER and the injured endosomes (Radulovic et al., 2022; Liu et al., 2023). At these contacts, the ORPs mediate the direct transfer of PS from the ER to the lysosomal membrane. The accumulated PS then activates the lipid transporter autophagy-related 2 homolog A (ATG2), which facilitates bulk lipid delivery for direct membrane repair. In a parallel pathway, the oxysterol-binding protein (OSBP) is similarly recruited by PtdIns4P to function as a redundant tether, exchanging cholesterol from the ER to stabilize the damaged site, thereby providing complementary structural support for membrane restoration (Zheng et al., 2025). In addition to ORPs and OSBP, extended synaptotagmin-1 (E-Syt1) is another critical tethering protein recruited by PtdIns4P. It constructs a more stable physical bridge between the ER and LEs, providing a structural platform for lipid transfer and potentially participating in the regulation of calcium signaling (Li et al., 2021; van Vliet et al., 2017).

## Endosome–mitochondria interactions: MCS-mediated cellular function

**Figure 5.**
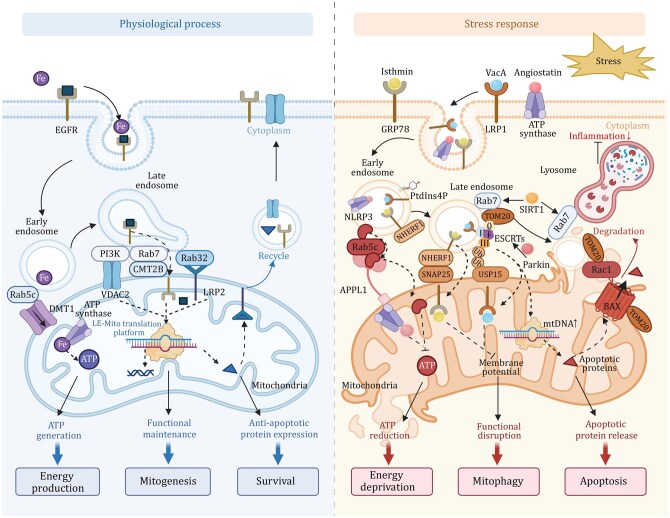
**The endosome-mitochondria interaction under normal or stress conditions**. The interaction between mitochondria and endosomes influences cell function. (i) Homeostatic regulation under physiological conditions: Upon activation of cell surface receptors (EGFR, ATP synthase), ATP synthase interacts with DMT1 in a Rab5c GTPase-coordinated manner, facilitating iron ion transport to enhance mitochondrial ATP synthesis and regulate energy production. As endosomes mature, PI3K and Rab32 form a translational platform with mitochondrial membrane proteins (VDAC2, CTM2B), promoting mitochondrial protein expression and mitogenesis. Concurrently, the Rab7-LRP2 interaction enhances the expression of anti-apoptotic proteins and sustains receptor recycling to support cell survival. (ii) Apoptotic activation under stress conditions: Internalized stress ligands (Isthmin, VacA, Angiostatin) show upregulated expression and bind GRP78/LRP1/ATP synthase before endocytosis. Early endosomes transport Angiostatin and the NLRP3 inflammasome to mitochondria, suppressing ATP production. Late endosomes, regulated by SNAP25 (mediating Isthmin) and USP15 (mediating VacA), induce mitochondrial membrane potential collapse and initiate apoptosis. TOM20 mediates Rac1-BAX binding to increase mitochondrial outer membrane permeabilization, releasing apoptotic proteins. Concurrently, TOM20 captures ubiquitinated proteins for lysosomal degradation. Endosome-dependent mitophagy is further promoted when stress-induced proteins like SIRT1 and Parkin activate Rab7, which drives the fusion of LEs and autophagosomes. Ultimately, mitophagy clears damaged mitochondria and degrades stress-related proteins, balancing cell survival and apoptosis. Abbreviations: EGFR: epidermal growth factor receptor; GRP78: glucose-regulated protein 78; Rab5c: Ras-related protein rab-5c; Rab32: Ras-related protein Rab-32; Rab7: Ras-related protein 7; Rac1: Rac family small GTPase 1; PI3K: phosphatidylinositol 3-kinase; CMT2B: Charcot-Marie-Tooth disease type 2B; VDAC2: voltage-dependent anion channel 2; DMT1: divalent metal transporter 1; VacA: vacuolating cytotoxin A; NLRP3: NLR family pyrin domain containing 3; TOM20: translocase of outer mitochondrial membrane 20; APPL1: adaptor protein phosphotyrosine interacting-like 1; BAX: BCL2-associated X protein; USP15: ubiquitin-specific peptidase 15; NHERF1: Na^+^/H^+^ exchanger regulatory factor 1; SNAP25: t-SNARE protein synaptosome-associated protein 25 kDa; NLRP3: NOD-like receptor family pyrin domain containing 3; LRP2: low-density lipoprotein receptor-associated proteins 2; LRP1: low-density lipoprotein receptor-associated proteins 1; ESCRTs: endosomal sorting complexes required for transports; SIRT1: sirtuin 1.This figure was created using BioRender.

Mitochondria are indispensable eukaryotic organelles that regulate diverse cellular functions spanning bioenergetic metabolism and cellular differentiation mechanisms (Zhang et al., 2018). Recent advances in organelle biology have revealed a sophisticated network of direct physical and functional interactions between mitochondria and endosomal compartments, fundamentally reshaping our understanding of cellular metabolic coordination and mitochondrial quality control (Fig. 5) (Todkar et al., 2019).

### Molecular basis of endosome–mitochondria interactions

The interaction between mitochondria and endosomes promotes the efficient shuttle of substances. Voltage-dependent anion channel 2 (VDAC2) is a mitochondrial outer membrane protein that interacts with components of phosphatidylinositol 3-kinase (PI3K) signaling to regulate endocytic transport processes through spatial regulation (Satoh et al., 2023). Small GTPase proteins play a key role in this mechanism, coordinating the bidirectional communication between mitochondria and endosomes. For example, Rac1 promotes translocation of effector proteins to mitochondria through cytoskeleton recombination; Rab5c, as an endosome regulator, participates in the regulation of mitochondria dynamics; Rab7 can be recruited to the mitochondria surface after sensing mitochondria damage to initiate mitophagy (Bailly et al., 2024; Yan et al., 2022); Rab32 transports anti-apoptotic proteins from EEs to mitochondria through interaction with low-density lipoprotein receptor-associated proteins 2 (LRP2, i.e. Megalin) to mitochondria (Daniele et al., 2014; Li et al., 2018, 2022).

It is worth noting that this interaction can facilitate the translocation of cell surface receptors to the mitochondria. For example, EGFR possesses this capability and can localize to mitochondria under specific conditions (Cao et al., 2011; Demory et al., 2009; Yao et al., 2010). The cytosolic scaffold protein Na^+^/H^+^ exchanger regulatory factor 1 (NHERF1), associated with LEs, and the t-SNARE protein synaptosome-associated protein 25 kDa (SNAP25) form a complex that facilitates lysosome-mitochondria interactions to transfer glucose-regulated protein 78 (GRP78) (Chen et al., 2014, 2018). In contrast, some typical mitochondrial proteins, such as ATP synthase (Chen et al., 2020b; Moser et al., 1999) and VDAC2, can also be released to the cell surface, performing receptor-like functions (De Pinto et al., 2010). In addition, divalent metal transporter 1 (DMT1) maintains iron homeostasis in mitochondria by directly transferring iron ions from EEs, thereby supporting oxidative phosphorylation (Barra et al., 2024). Lipid transport also plays a vital role in the contact mechanism. PDZD8 can form a tripartite junction between the ER, mitochondria, and endosomes, which is crucial for cholesterol transfer and endosome maturation. Its dysfunction can exacerbate lipotoxicity in podocytes (Elbaz-Alon et al., 2020; Hasegawa et al., 2024). A recent study demonstrates that SIRT1 interacts with Rab7 to promote the selective elimination of damaged mitochondria in lung endothelial cells in sepsis-induced acute lung injury (Jiang et al., 2024). Endosomal dysfunction may exacerbate mitochondrial damage in alveolar epithelial cells, contributing to the pathogenesis of this life-threatening condition (Jiang et al., 2024).

### Physiological function of mitochondria quality control

To efficiently remove damaged mitochondrial components before they cause cellular harm or trigger inflammation, cells employ several quality-control pathways (Hasegawa et al., 2024). These quality control mechanisms include mitophagy (Hasegawa et al., 2024), sequestration into endosomes (Hamacher-Brady and Brady, 2016), and the degradation of selective mitochondrial content using LEs (Soubannier et al., 2012; Sugiura et al., 2014). Significantly, studies in murine models have shown that loss of either protein, such as PINK1 and Parkin, impairs the autophagic clearance of damaged mitochondria, which can trigger a STING-dependent inflammatory response (Sliter et al., 2018). This demonstrates that PTEN-induced kinase 1 (PINK1) and Parkin help regulate immune activation by controlling lysosomal degradation pathways for defective mitochondria (Pickrell and Youle, 2015). Under stress conditions, Parkin is recruited to the outer mitochondrial membrane, where it ubiquitinates proteins. The ubiquitin-tagged mitochondrion is then recognized and captured by ESCRT complexes on the surface of an early endosome. The ESCRT machinery drives the invagination and scission of the endosomal membrane, ultimately internalizing the mitochondrion within the endosomal lumen (Hammerling et al., 2017). Furthermore, damaged mitochondria can release mitochondrial DNA (mtDNA) into the cytosol, where it acts as a damage-associated molecular pattern (DAMP) and triggers a STING-dependent type I interferon response (West et al., 2015).

Notably, mitochondria are positioned at these endosome-mediated translation sites, where mRNA for mitochondrial proteins is translated directly on the surface of LEs. In neuronal axons, LEs act as platforms for mRNAs—including those encoding mitochondrial proteins—and the local translation of these transcripts is essential for mitochondrial maintenance. Supporting this, local translation of Lamin B2 mRNA, an intermediate filament protein typically associated with the nuclear membrane, was found to sustain axonal health by promoting mitochondrial function (Yoon et al., 2012). These findings suggest that LEs may serve as key subcellular hubs for compartmentalized translation, thereby supporting mitochondrial homeostasis, while mitochondria themselves may help facilitate activity-dependent translational plasticity. These RNA-carrying vesicles interact with ribosomes and transiently dock onto mitochondria in retinal ganglion cell axons, forming a localized translation center that maintains mitochondrial function (Liang, 2021). However, preclinical studies suggest that Rab7 mutations linked to Charcot-Marie-Tooth disease type 2B (CMT2B) disrupt localized protein synthesis, impair mitochondrial homeostasis, and ultimately compromise axonal integrity (Cioni et al., 2019).

### Pathological implications of stress response and apoptosis

Extracellular Angiostatin and Isthmin can be transported into mitochondria after endothelial cell endocytosis, disrupting mitochondrial function and mediating cell apoptosis (Chen et al., 2014; Moser et al., 1999). In addition, the Vacuolating cytotoxin A (VacA) of Helicobacter pylori can induce apoptosis via LRP1, thereby exerting its toxic effect (Calore et al., 2010). The endosomal compartment plays a key role in this process, acting as a central hub that curates the removal of damaged mitochondrial components before triggering oxidative stress or inflammatory cascades (Hammerling et al., 2017; Tábara et al., 2025). On the one hand, Rac1 can promote internal volume accumulation in apoptotic mitochondria (Bailly et al., 2024), thereby driving increased mitochondrial outer membrane permeability, assembling functional BCL-2-associated X protein (Bax) pores, and facilitating the release of pro-apoptotic factors (Vicinanza and Rubinsztein, 2020). Although knockdown of Rab5c or ubiquitin-specific peptidase 15 (USP15) mainly affects Bax activation, Rab5c depletion impairs the mitochondrial recruitment/activation of BAX (Gordaliza-Alaguero et al., 2025; Ravindran et al., 2022; Vicinanza and Rubinsztein, 2020).

Meanwhile, a new regulatory network between EEs and mitochondria can control the activation of NLRP3 inflammasomes (Lee et al., 2023; Wu and Cheng, 2022). Pathogens or cellular stress trigger the accumulation of PtdIns4P in EE, which directly recruits NLRP3 to endosomal surfaces, thereby driving inflammasome assembly (Zhang et al., 2023). NLRP3 agonist can induce the transfer of the adaptor protein, phosphotyrosine interaction, PH domain, and leucine zipper containing 1 (APPL1) from EEs to mitochondria, where it interacts with Rab5, promoting mitophagy (Hammerling et al., 2017; Ryan et al., 2020; Wu et al., 2021). Meanwhile, sirtuin 1 (SIRT1) activates Rab7 protein to promote fusion between LEs and autophagosomes, forming endosome-dependent mitophagy. This process thereby suppresses excessive activation of both the NLRP3 and the cytosolic nucleotide-sensing STING pathways (Jiang et al., 2024). On the other hand, TOM20 (translocase of the outer mitochondrial membrane complex subunit 20)-positive vesicles in damaged mitochondria are upregulated and interact with ubiquitin-tagged mitochondrial proteins and endosomal membranes to ensure that they are transported to the lysosomes for degradation (Soubannier et al., 2012).

## Endosome–nucleus interactions: signaling and mechanical coupling

Endosomes are traditionally viewed as sorting hubs for membrane trafficking. Still, recent studies have revealed their role as a multifunctional signaling platform, establishing direct or indirect molecular dialogue with the nucleus through multiple pathways (Chaumet et al., 2015). This bidirectional communication mechanism regulates gene expression and cell fate, participating in essential biological processes such as cell cycle regulation and stress response.

### Molecular basis of endosome-nucleus communication

The ESCRT complex packages activated receptors or transcription factors by generating intraluminal vesicles (ILVs), forming signaling carriers that are transported to perinuclear regions (Migliano and Teis, 2018). The ESCRT-III component directly participates in nuclear envelope rupture repair, with its recruitment dependent on damage sensing by LEM-domain proteins (Olmos et al., 2015). ESCRT-I/II mediates the clearance of misassembled nuclear pore complexes through recognition mechanisms, thereby maintaining nucleocytoplasmic barrier integrity. The involvement of ESCRT in both endosomal ILV formation and nuclear envelope repair reveals its core role as a versatile membrane-remodeling machinery. The LINC complex (linker of nucleoskeleton and cytoskeleton) may also serve as a key protein machinery mediating contact between the nucleus and endosomal membranes. Through their interaction with cytoskeletal elements, KASH (Klarsicht, ANC-1, Syne Homology) proteins on the outer nuclear membrane could provide docking sites for endosomes traveling along the cytoskeleton, facilitating their anchoring at the nuclear periphery. Conversely, SUN (Sad1 and UNC-84 domain) proteins may transmit mechanical or chemical signals from perinuclear endosomes into the nuclear interior, potentially influencing gene expression (Cain et al., 2018). Future studies aimed at identifying specific endosomal binding partners of KASH proteins and elucidating the functional consequences of these contacts will be essential for understanding this emerging interface in inter-organelle communication (Eriksson et al., 2003). Rab5-positive endosomes form signaling hubs dynamically interacting with perinuclear regions by recruiting APPL1/2 adaptor proteins (Miaczynska et al., 2004). During mitosis, Rab5 endosomes regulate spindle orientation and nuclear envelope disassembly by controlling the cortical localization of the Mud protein, underscoring their critical role in coordinating cell cycle progression (Capalbo et al., 2011). Mutations in nuclear envelope structural proteins, particularly LMNA encoding Lamin A/C, lead to nuclear membrane instability and profound defects in nuclear architecture. Genetic studies have linked over 500 mutations in LMNA to a spectrum of human diseases collectively termed laminopathies, including muscular dystrophies, cardiomyopathies, and premature aging syndromes such as Hutchinson-Gilford progeria syndrome (HGPS) (Zhou and Song, 2025). Pseudomonas exotoxin A (PE) exploits the low motility and fusion-permissive characteristics of nuclear-associated endosomes to directly release toxic components into the perinuclear space, revealing a novel pathogenic strategy that hijacks endosome-nuclear communication (Chaumet et al., 2015). Lipid signaling plays a pivotal regulatory role. PtdIns(4,5)P_2_ modulates nuclear transcription factors through PI3K-AKT pathway activation, while its effector proteins (including SNX family members) coordinate endosomal maturation with nuclear trafficking (Tan et al., 2015). PtdIns3P enrichment on endosomal membranes recruits FYVE/RBX effector proteins to initiate autophagosome biogenesis. Nuclear PtdIns3P pools may indirectly influence chromatin dynamics through the mTORC1-ATG signaling axis, though direct chromatin remodeling evidence remains limited (Di Paolo and De Camilli, 2006; Hong et al., 2017; Nascimbeni et al., 2017).

### Physiological function of nuclear signaling and gene regulation

Endosomes regulate nuclear transcriptional activities by transporting signaling molecules to activate downstream kinase cascades. Following internalization, the epidermal growth factor receptor sustains extracellular signal-regulated kinases 1/2 (ERK1/2) activation, promoting their translocation into the nucleus to drive cell proliferation through phosphorylation of transcription factors such as c-Fos/c-Jun (Oyarzún et al., 2014). Similarly, the transforming growth factor-β receptor activates SMAD complexes via endosomes to modulate the expression of genes related to fibrosis and differentiation. Notably, selective inhibition of endosomal Gαs signaling significantly reduces nuclear ERK activity, MYC expression, and cell proliferation (Kwon et al., 2022). Studies on GPCR signaling reveal that MVB-localized receptors (e.g., Frizzled) or components activate β-catenin nuclear translocation, a mechanism involving molecular interaction and recruitment of low density lipoprotein receptor-related proteins 5 and 6 (LRP5 and LRP6) (Dobrowolski et al., 2012; Mills, 2012). Pharmacological blockade of clathrin-mediated endocytosis and dynamin GTPase activity blocks calcitonin receptor-like receptor (CLR) endocytosis and associated signaling, including cytosolic protein kinase C (PKC) activation and nuclear ERK stimulation, which originates from endosome-localized CLR trafficking events (Yarwood et al., 2017). Nuclear targeting mechanisms of retrograde trafficking pathways have been elucidated. The COP-I vesicle complex enables receptor tyrosine kinases (RTKs) to enter the nucleus via the endosome-Golgi-ER pathway, establishing sustained pro-proliferative signals through the coordinated action of Sec61 translocation and Importin-mediated nuclear transport systems (Chen et al., 2020b).

Endosome-localized nucleocytoplasmic proteins also regulate nuclear transcription. For example, the DNA repair protein Ku70 forms a ternary complex with Ras/Raf in the cytoplasm, anchoring to Rab5/Rab7 positive endosomes. Studies suggest that this complex may suppress tumorigenesis by inhibiting the Cdc25A (cell division cycle 25A)-CDK1 (cyclin-dependent kinase 1) cell cycle axis via the MEK (mitogen-activated extracellular signal-regulated kinase)-ERK pathway (Pandey et al., 2024). Certain endosomal cargoes generate nuclear-localized fragments through proteolytic cleavage for direct nuclear entry. The notch receptor releases its intracellular NICD domain upon endosomal cleavage, enabling its nuclear translocation to activate target genes such as Hes1 (Borggrefe et al., 2016). Similarly, FGFR4 (fibroblast growth factor receptor 4) forms a complex with Src/STAT3 in endosomes, which co-translocates to the nucleus to regulate gene expression (Shin and Ahn, 2021). HGF-MET signaling drives erythropoietin-producing hepatocellular A2 (EphA2) endosomal trafficking via a Rab17-clathrin-dependent pathway, altering nuclear actin dynamics through the RhoG-cofilin axis to activate the MRTF/SRF transcriptional program, thereby promoting tumor cell invasion (Marco et al., 2021). Nuclear-periphery endosomes are involved in the localized synthesis of PtdIns3P and PtdIns(3,4)P_2_, which recruits the PLEKHA7/β-catenin complex to enable compartmentalized activation of signaling pathways (Schink et al., 2013; Tan et al., 2015). Beyond their canonical endocytic roles, the non-canonical nuclear functions of endocytic regulators—including Eps15, Epsin1, APPL1/2, and the ESCRT machinery—introduce multi-compartment complexity to cellular signaling networks governing cell cycle progression, proliferative outputs, and metabolic homeostasis (Pilecka et al., 2007). Recent advances have expanded the concept of cellular signaling from a cytosol-centered approach to a parallel nuclear signal transduction (Carrillo et al., 2024; Chen et al., 2020a, 2022a, 2025a; Choi et al., 2019; Hou et al., 2025b; Wen et al., 2025). Thus, endosome–nucleus interactions may bridge the cytosol and nucleus, representing a sophisticated communication network that integrates extracellular cues with genomic responses.

## Endosome–cytoskeleton interactions: mechanical coupling

The spatiotemporal orchestration of intracellular trafficking and cellular motility hinges on the dynamic interplay between endosomal compartments and cytoskeletal networks (actin filaments and microtubules) (Xiang et al., 2015). This intricate coordination enables precise regulation of cargo transport, membrane remodeling, and signaling cascades through distinct yet interconnected mechanisms.

### Molecular basis of trafficking

Molecular mechanisms in trafficking endosomes employ actin filaments for short-range maneuvers (<5 μm) and microtubules (MTs) for long-distance transport (>10 μm). Actin drives critical processes at the plasma membrane interface. Arp2/3-mediated polymerization facilitates endocytic vesicle scission (Toshima and Toshima, 2024). Myosin V/VI motors mediate vesicle anchoring and actin-track-based transport (Seaman et al., 2013). The Rho GTPase activating protein 22 (ARHGAP22) emerges as a critical modulator of actin dynamics at EEA1/Rab11-positive endosomes, modulating actin dynamics by inactivating Rac1/Cdc42 at EEA1/Rab11-positive endosomes (Mori et al., 2014). During endosomal maturation, EEs undergo a spatial transition from peripheral actin-dependent movements to MT-based trajectories, regulated by Rab GTPase cascades. Rab5 orchestrates early endocytic processes through four distinct functions: (i) targeting endocytic vesicles to endosomes; (ii) mediating early endosome fusion; (iii) modulating endosomal motility; and (iv) initiating retrograde transport via recruitment of dynactin-dynein complexes (Bucci et al., 1992; Gorvel et al., 1991; Horiuchi et al., 1995; McLauchlan et al., 1998; Toshima and Toshima, 2024). The recruitment of specific motors to endosomal subdomains is governed by a combination of luminal cues and cargo-specific factors. For instance, the small GTPase Rab7 recruits the dynein/dynactin complex via its effector, RILP (Rab-interacting lysosomal protein), facilitating minus-end-directed movement toward the perinuclear region (Rink et al., 2005; Toshima and Toshima, 2024). Moreover, changes in luminal pH (mediated by the V-ATPase) serve as a critical cue. This pH-shift activity of transmembrane proteins and associated small GTPases, which in turn creates docking platforms for motor adaptors. Concurrently, the dynamic conversion of phosphoinositide species serves as a molecular address code that facilitates the recruitment of dynein (Fares et al., 2025; Renard et al., 2018). Notably, the recently characterized commander complex regulates endosomal maturation and cargo sorting, thereby indirectly influencing cytoskeletal remodeling (Yong et al., 2023).

Phosphoinositide signaling further facilitates endosomal trafficking along the cytoskeleton, contributing to its diverse regulatory roles in cell motility, stress adaptation, immune response, oncogenesis, and metastasis (Hou et al., 2025a, 2025c; Ke et al., 2024; Ren et al., 2024, 2025; Sun and Chen, 2026; Tang et al., 2026). For example, microtubule-associated protein 4 (MAP4) directs endosomal class I PI3Kα localization to generate phosphoinositide PtdIns(3,4,5)P_3_ to activate PI3K-AKT signaling by binding its microtubule-binding domain to the C2 domain of the PI3K p110α catalytic subunit (Thapa et al., 2020). MAP4 is essential for PI3Kα interaction with activated receptors on endosomes, and its loss disrupts PI3K-AKT signaling downstream of multiple agonists (Sun and Chen, 2026; Thapa et al., 2020). Upon agonist stimulation or knockdown of the PI3K regulatory subunit p85α, residual p110α—mainly coupled to p85β—shows enhanced recruitment to receptor tyrosine kinases on endosomes (Thapa et al., 2024). The p110α C2 domain also binds phosphoinositide PtdIns3P, which is required for endosomal targeting and PI3K-AKT signaling (Thapa et al., 2024). Stable p85α knockdown, mimicking cancer-associated reduction, increases cell growth and tumorsphere formation; these effects are reversed by MAP4 or p85β knockdown, highlighting the role of endosome–cytoskeleton interactions in promoting cell motility and oncogenic signaling (Chen et al., 2025b; Hou et al., 2025c; Thapa et al., 2024).

### Dynamic regulation of motor proteins

Motor protein coordination drives bidirectional endosomal transport along MTs (Bielska et al., 2014; Welte, 2004; Xiang et al., 2015). The minus-end-directed dynein motor mediates centripetal transport through dynactin-mediated processivity enhancement and Hook protein adaptors (Canty et al., 2021; Paschal et al., 1987; Schroer et al., 1989). Notably, the WD-repeat protein lissencephaly-1 (LIS1) regulates dynein motility by stabilizing its MT attachment without altering ATPase activity (Huang et al., 2012). Hook proteins demonstrate dual functionality, simultaneously recruiting kinesin-3 to early endosomes while coordinating dynein activity, suggesting a scaffold-based mechanism for motor coordination (Fu and Holzbaur, 2014). The anterograde transport machinery employs kinesin-1, which mediates long-range anterograde transport, while kinesin-3 family members drive short-range, processive movements to the periphery (Bielska et al., 2014). Remarkably, single dynein molecules can override multiple kinesin-3 motors through a phosphorylation-regulated tug-of-war mechanism (Canty et al., 2021; Hirokawa et al., 2009). This bidirectional control is further modulated by Rab effectors (e.g., Rab6) and post-translational modifications (Grant and Donaldson, 2009; Hirokawa et al., 2009; Xiang et al., 2015), as kinesins function as phosphoproteins with activity states responsive to cellular signaling (Hollenbeck, 1993; Sato-Yoshitake et al., 1992).

Beyond simple transport, these motors generate the mechanical forces necessary for membrane tubulation and fission. During tubular-based sorting, the simultaneous pulling of motors anchored on the endosomal membrane creates tension. This force extracts lipid tubules from the endosome core, effectively segregating recycling cargo from degradative cargo. The subsequent fission of these tubules often requires coordination of motor-generated tension with the scission machinery, such as ESCRT-III or dynamin-related proteins (Renard et al., 2018). A failure in this coordination leads to elongated endosomal tubules that fail to detach, thereby disrupting protein sorting and cellular homeostasis (Bhattacharyya and Pucadyil, 2024).

### Physiological function of cell motility and invasion

During chemotaxis, recycling endosomes utilize kinesin-driven MT transport to polarize integrin/EGFR delivery to the leading edges, thereby facilitating focal adhesion turnover and membrane protrusion (Grant and Donaldson, 2009). Actin-associated myosin V/VI motors fine-tune this process by enabling polarized secretion of matrix metalloproteinases (e.g., MT1-MMP) at invadopodia (Linder et al., 2023). During malignant progression, this secretory pathway is amplified by oncogenic Rab25-retromer-Wnt/Notch signaling axes (Seaman et al., 2013; Toshima and Toshima, 2024). Conversely, LIS1 mutations disrupt dynein-mediated endosome-nucleus communication, thereby impairing neuronal migration during development (Reiner et al., 1993; Xiang et al., 2015).

## Future perspectives and technical limitations

Endocytosis is not merely a passive mechanical transport process; rather, it serves as a dynamic coordinator of cellular activities through membrane contacts (Table 1). These contacts facilitate interactions with various organelles, including lysosomes, the Golgi apparatus, mitochondria, the ER, the nucleus, and the cytoskeleton (Cullen and Steinberg, 2018; Park et al., 2021; Rocha et al., 2009; Todkar et al., 2019; Wenzel et al., 2022). By mediating fusion events, these contact sites enable material transport, signal regulation, and metabolic control, thereby playing a crucial role in maintaining global cellular homeostasis (Table 2).

**Table 1.** Core molecular machinery of endosomal homeostasis.

Functional category	Representatives	Main functions	Related internal area rooms
Tethers and SNAREs	EEA1, STXs, VAMPs	Mediate endosome-endosome homotypic fusion and endosome other-organelle heterotypic fusion	EE, LE, Lysosome
Sorting complexes	Retromer, ESCRTs, BLOCs	Mediate cargo sorting, degradation, and lysosome-associated organelle generation	EE, LE, MVBs
Rab GTPases	Rab5, Rab4, Rab6, Rab7, Rab9, Rab11, Rab22, Rab25, Rab32, Rab38	Define internal identity and recruit downstream effectors (motors, tethering factors)	EE, RE, LE
Motor adaptors	RILP (Rab7), FYCO1 (Rab7), ORP1L	Connect the endosome and the cytoskeleton motor to regulate transport direction and speed	LE (RILP), EE/LE (FYCO1)
Lipid metabolizing enzymes and transfer proteins	Vps34 (class III PI3K), PI4K2A PIKfyve, class I PI3Ks, OSBP, ORPs	Generate, convert, and transfer lipids to provide docking sites for sorting complexes, signaling, and membrane repair	EE, LE, Lysosome
Phosphoinositide signal	PtdIns3P, PtdIns4P, PtdIns(4,5)P_2_, PtdIns(3,4)P_2_, PtdIns(3,4,5)P_3_	Identify dynamic membrane sign and facilitate signal integration, transduction, and amplification	EE, LE, Lysosome

**Table 2.** Endosomal interorganellar contact sites: cellular functions and homeostasis.

Endosomal contact organelle	Key molecules	Main functions	Stress/Metabolic regulation
Lysosome	BLOCs, HOPS complex, ESCRTs	Preparation before fusion, mixing of contents, and regeneration of lysosomes	Enhance contact during nutrient deficiency, promote autophagy flow, and degradation product recovery
Golgi	Retromer (AP-1, AP-3, GGAs, Clathrin, and ARF1), ESCPE-1	Recycling receptors (such as M6PR) from the endosome back to the Golgi apparatus	Regulating recycling flux and maintaining lysosomal enzyme supply during nutritional stress
Endoplasmic reticulum	VAPs, PDZD8-Protrudin, STARD3, RNF26, MOSPD2, PTP1B	Endosomal localization regulation, cholesterol/lipid transport, and Ca²+ exchange	Enhance exposure to promote cholesterol intake during low cholesterol levels; Ca²+ signal regulates autophagy initiation
Mitochondria	DMT1, VDAC2, APPL1, CMT2B, LRP2, SNAP25	Iron sulfur cluster transfer, and membrane contact point regulation of cellular metabolism and stress	Enhanced contact under oxidative stress may mediate the delivery of damaged mitochondria to autophagosomes
Nucleus	ESCRTs, LINC complex	Signal transduction and gene expression regulation	Perinuclear aggregation of endosomes under stress may regulate transcription factor activity
Cytoskeleton	Kinesins, dynein/dynactin, and their adaptors	Simple transport, membrane tubulation, and fission.	Stress promotes cell motility and oncogenic signaling

As research progresses, it has become increasingly evident that specific disruptions in endosomal contact sites are closely linked to a range of organelle-related diseases. For instance, endosomal-lysosomal fusion is essential for waste degradation, and defects in this process are associated with conditions such as hereditary spastic paraplegia (Gros and Muller, 2023). Strategies aimed at inhibiting Rab7 are currently being explored to broadly downregulate hyperactive endocytic pathways in neurodegenerative disorders (Jordan et al., 2022). Moreover, deficiencies in these complexes have been implicated in the development of neurodegenerative diseases, tumors, diabetes, and metabolic syndromes (Gros and Muller, 2023; Sun et al., 2026). For example, inhibitors of the class III PI3K Vps34, such as SAR405, are under investigation for their ability to block autophagy and sensitize tumors to immune checkpoint blockade by modulating the endosomal trafficking of PD-L1 and STING (Lee et al., 2024). In parallel, Rab11, a master regulator of recycling endosome function, controls the surface expression of multiple pro-metastatic cargoes, including integrins and matrix metalloproteinases (MMPs). In aggressive cancers, oncogenic signaling hyperactivates Rab11-dependent recycling, maintaining high surface levels of these cargoes and promoting cell migration and invasion. Preclinical studies have shown that allosteric Rab11 inhibitors can disrupt recycling of these oncogenic cargoes, reducing metastatic potential in xenograft models without affecting general endosomal housekeeping functions. This node-specific targeting, focusing on the Rab11 effector interaction interface, exemplifies the concept of a “mechanistically actionable” intervention, in which a detailed understanding of a specific molecular node enables selective therapeutic targeting (Lempicki et al., 2024). In the context of neurological disorders, endocytic pathways influence the reassembly of key proteins within the Golgi apparatus (Vardarajan et al., 2012). Such disruptions may lead to abnormal dendritic morphology and contribute to disease pathogenesis. Notably, three proteins involved in endosomal-Golgi interactions, the AP-5/SPG11/SPG48 complex, have been identified as critical factors in the pathogenesis of hereditary spastic paraplegia. Moreover, inhibiting mitochondrial fission has been shown to underlie both cytoskeletal disorganization and axonal degeneration in SPG11 and SPG48 neurons in a preclinical study, highlighting the therapeutic potential of targeting these pathological mechanisms; indeed, such inhibition has been demonstrated to rescue neuronal degeneration in models of hereditary spastic paraplegia (Chen et al., 2022b). However, clinical intervention with Miglustat does not appear to alter disease progression in clinical use (Mero et al., 2025). In AD experimental models, defective endosomal trafficking contributes to impaired mitophagy/QC, thereby promoting disease progression (Blackstone, 2018; Pickrell and Youle, 2015). Similarly, in diabetic nephropathy experimental models, endosomal dysfunction may impair the reabsorption of glucose and amino acids by renal tubular cells, contributing to tubular injury (Tagliatti and Cortese, 2022). Inhibition of the sodium-glucose cotransporter 2 (SGLT2) by dapagliflozin in a preclinical study has been shown to ameliorate tubular proteinuria and tubulointerstitial injury in the early stages of diabetic kidney disease (Farias et al., 2023). Despite promising perspectives for targeting endosome-related pathogenesis, first-line clinical research in this area still requires further investigation.

Although the core machinery that governs endosomal trafficking is evolutionarily conserved, the functional architecture and dynamic behavior of these intracellular hubs display remarkable cell-type specificity. It is therefore important to recognize that the canonical model of endosomal function, largely derived from studies in non-polarized cells, may not fully capture the physiological nuances inherent to specialized cell types. In highly polarized neurons, endosomal hubs must coordinate spatially segregated tasks. Endosomes are trafficked over long distances along the axon, retrogradely toward the cell body via dynein motors and anterogradely toward the distal axon via kinesins. It could support processes such as neurotransmitter receptor turnover and neurotrophic signaling (Cosker and Segal, 2014). In polarized epithelial cells, by contrast, these hubs are essential for the asymmetric sorting of membrane proteins required to establish and maintain apical-basal polarity. Distinct populations such as apical EEs, basolateral EEs, and at least two functionally distinct REs—apical REs and common REs—ensure that internalized transmembrane proteins are properly sorted for recycling to the cell surface, lysosomal degradation, transcytosis, or retrograde transport to the TGN, thereby preserving barrier function and directed secretion (Cao et al., 2012; Fölsch et al., 2009). Even in immune cells, endosomal compartments undergo specialization, facilitating the fusion of phagosomes with endosomes and the formation of antigen-processing compartments (Mayorga et al., 1991). Thus, integrating this cell-type-specific context is critical for a comprehensive understanding of endosomal hub functions in both physiology and disease. Recent advancements in imaging technologies, such as proximity ligation assay, cryo-electron tomography, and optogenetic tools, have significantly enhanced our understanding of the protein composition of MCSs (Carette et al., 2011; Chen et al., 2021; Van Engelenburg et al., 2014). However, most studies to date have relied on simplified models that fail to account for tissue-specific or stress-induced heterogeneity and often lack the complexity of *in vivo* microenvironments. As a result, our comprehension of the intricate interactions between different MCSs remains limited.

Looking ahead, further progress in the study of MCSs will hinge on the integration of diverse technological approaches. Representative advancements include: (i) haploid genetic screens that have already defined critical roles for NPC1 cholesterol transporters and the HOPS complex in filoviral escape from host endocytic trafficking systems (Gong et al., 2016); (ii) Super-resolution microscopy platforms enabling nanoscale mapping of virion-endosome interface dynamics (Lu et al., 2019). However, the resolution limit of conventional light microscopy makes it challenging to definitively distinguish between true molecular contact and mere close apposition. Therefore, while the functional data supporting these interactions is robust, the structural interpretation of imaging data should be made with caution; (iii) Organelle-restricted biosensor arrays for real-time visualization of intercompartmental interactomes (Shao et al., 2021); (iv) CRISPR-based functional genomics for systematic identification of endosomal signaling regulators (Lu et al., 2022; Uijttewaal et al., 2025); (v) Smart nanocarrier systems engineered to disrupt pathogenic cross-talk through endosomal-mitochondrial axis modulation, exemplified by therapeutic strategies targeting pathological protein oligomerization (Bhosle et al., 2019). Collectively, these synergistic approaches will elucidate the fundamental principles of cellular communication networks, enabling precise intervention in endosome-related pathologies.

## Conclusions

Endosomes function as dynamic signaling hubs that coordinate cellular homeostasis through extensive membrane contact networks with lysosomes, the Golgi, mitochondria, the endoplasmic reticulum, the nucleus, and the cytoskeleton. Disruption of these interorganellar interactions contributes to diverse disease pathologies, prompting the development of therapeutic strategies targeting key endosomal regulators. Nevertheless, clinical translation remains challenging. Advancing our understanding will require integrating super-resolution imaging, organelle-targeted biosensors, and CRISPR-based screens to distinguish true membrane contact from close apposition and to elucidate tissue-specific heterogeneity. Such multidisciplinary approaches will ultimately enable precise therapeutic interventions in endosome-associated diseases.

## Artificial intelligence (AI)

During the preparation of this manuscript, the author used ChatGPT to improve clarity and narrative flow. All outputs were carefully reviewed and edited as necessary, and the author assumes full responsibility for the accuracy, integrity, and final content of the published article.

## Abbreviations

AD: Alzheimer’s disease
ALIX: ALG-2-interacting protein X
APP: amyloid precursor protein
APPL1: adaptor protein, phosphotyrosine interaction, PH domain, and leucine zipper containing 1
ARHGAP22: Rho GTPase activating protein 22
ASGPR: asialoglycoprotein receptor
ATG2: autophagy-related 2 homolog A
Aβ: amyloid-β
BAR: Bin-Amphiphysin-Rvs
BLOCs: biogenesis of lysosome-related organelle complexes
BORC: BLOC-1-related complex
Cdc25A: cell division cycle 25A
CDK1: cyclin-dependent kinase 1
CGN: cis-Golgi network
CIMPR: cation-independent mannose 6-phosphate receptor
CLR: calcitonin receptor-like receptor
CMT2B: Charcot-Marie-Tooth disease type 2B
CORVET: class C core endosomal vacuole tethering
cPLA2α: cytosolic phospholipase A2α
DAMP: damage-associated molecular pattern
DMT1: divalent metal transporter 1
EEA1: early endosome antigen 1
EEs: early endosomes
EGFR: epidermal growth factor receptor
EphA2: erythropoietin-producing hepatocellular A2
ERK1/2: extracellular signal-regulated kinases 1/2
ESCRT: endosomal sorting complexes required for transport
ESCPE-1: endosomal SNX-BAR sorting complex for promoting exit 1
E-Syt1: extended synaptotagmin-1
FAM21: family with sequence similarity 21
FGFR4: fibroblast growth factor receptor 4
GARP: Golgi-associated retrograde protein
G-CSFR: granulocyte colony-stimulating factor receptor
GEF: guanine nucleotide exchange factor
GGAs: Golgi-localized γ-ear-containing ARF-binding proteins
GMII: Golgi α-mannosidase II
GPCRs: G protein-coupled receptors
GRP78: glucose-regulated protein 78
HGPS: Hutchinson-Gilford progeria syndrome
HOPS: homotypic fusion and protein sorting
HPS: Hermansky-Pudlak syndrome
IGF2R: insulin-like growth factor 2 receptors
ILVs: intraluminal vesicles
IP3R: inositol 1,4,5-trisphosphate receptor
IST1: increased sodium tolerance 1
KASH: Klarsicht, ANC-1, Syne Homology
KIF13A: kinesin family member 13A
LBPA: lysobisphosphatidic acid
LEs: late endosomes
LINC: linker of nucleoskeleton and cytoskeleton
LIS1: lissencephaly-1
LMP: lysosomal membrane permeabilization
LPA: lysophosphatidic acid
LRP2: low-density lipoprotein receptor-associated proteins 2
LROs: lysosome-related organelles
LRRK1: leucine-rich repeat kinase 1
M6PR: mannose-6-phosphate receptor
MAP4: microtubule-associated protein 4
MCS: membrane contact site
MEK: mitogen-activated extracellular signal-regulated kinase
MMPs: matrix metalloproteinases
MOSPD2: motile sperm domain-containing protein 2
mtDNA: mitochondrial DNA
MTs: microtubules
MVBs: multivesicular bodies
NHERF1: Na^+^/H^+^ exchanger regulatory factor 1
NLRP3: NOD-like receptor family pyrin domain-containing 3
NPC1: Niemann-Pick C1 protein
ORP: OSBP-related protein
OSBP: oxysterol-binding protein
PA: phosphatidic acid
PDGFR: platelet-derived growth factor receptor
PE: Pseudomonas exotoxin A
PI3K: phosphatidylinositol 3-kinase
PINK1: PTEN-induced kinase 1
PKC: protein kinase C
PS: phosphatidylserine
PtdIns(3,5)P2: phosphatidylinositol-3,5-bisphosphate
PtdIns3P: phosphatidylinositol 3-phosphate
PtdIns4P: phosphatidylinositol 4-phosphate
PTP1B: protein-tyrosine phosphatase 1B
REs: recycling endosomes
RILP: Rab-interacting lysosomal protein
RNF13: RING finger protein 13
RNF26: Ring Finger Protein 26
RTKs: receptor tyrosine kinases
SGLT2: sodium-glucose cotransporter 2
SIRT1: sirtuin 1
SNAP25: synaptosome-associated protein 25 kDa
SNARE: soluble N-ethylmaleimide-sensitive factor attachment protein receptor
SNX: sorting nexin
SORL1: sorting receptor sortilin-related receptor 1
SQSTM1: sequestosome 1
STARD3: StAR-related lipid transfer domain-containing 3
SUN: Sad1 and UNC-84 domain
TGN: trans-Golgi network
TIP47: tail-interacting protein of 47 kilodaltons
TMCC1: transmembrane and coiled-coil domain family 1
TOM20: translocase of the outer mitochondrial membrane complex subunit 20
TPC1: two-pore cation channel 1
TRPML1: transient receptor potential mucolipin 1
USP15: ubiquitin-specific peptidase 15
VDAC2: voltage-dependent anion channel 2
